# A comparison of malaria prevention behaviours, care-seeking practices and barriers between malaria at-risk worksite migrant workers and villagers in Northern Shan State, Myanmar—a mixed method study

**DOI:** 10.1186/s12936-022-04193-8

**Published:** 2022-06-03

**Authors:** Ye Kyaw Aung, Su Su Zin, Kemi Tesfazghi, Mahesh Paudel, May Me Thet, Si Thu Thein

**Affiliations:** 1 Strategic Information Department, Population Services International, 16, West Shwe Gone Dine 4th Street, Bahan Township, Yangon, Myanmar; 2 Population Services International, Washington, DC USA

**Keywords:** Migrant workers, Malaria prevention behaviors, Care-seeking practices, Worksites, Myanmar

## Abstract

**Background:**

Migrant populations are at an increased risk of exposure to malaria due to their nature of work and seasonal migration. This study aimed to compare malaria prevention behaviours and care-seeking practices among worksite migrant workers and villagers in the malaria-at-risk areas of Eastern Myanmar close to the China border.

**Methods:**

A mixed method study was conducted in March 2019. The malaria-at-risk worksites in the four targeted townships, and villages located the nearest to these worksites were approached. Key stakeholders, such as worksite managers and village leaders, were interviewed.

**Results:**

A total of 23 worksites, which employed 880 migrants and 447 locals, and 20 villages, which were homes for 621 migrants and 9731 locals, were successfully interviewed. Regarding malaria prevention behaviours, sleeping under a bed net was common among both worksites (74%) and villages (85%). In contrast, insecticide-treated nets/long-lasting insecticidal nets (ITN/LLIN) usage was much lower in the worksites than in the villages (39% vs 80%). Regarding care-seeking practices for febrile illness, self-medication was a popular choice for both worksite workers and villagers owing to the easy availability of western medicine. Moreover, local-belief-driven traditional practices were more common among villagers. For occasions in which fever was not relieved, both would seek health care from rural health centres, private clinics, or public hospitals. As for barriers, villagers mostly cited language barriers, which often lead to misunderstanding between health providers and them. In contrast, most of the worksites cited logistics issues as they were in remote areas with devastated road conditions and the routes to formal health facilities were not secure due to frequent armed conflicts.

**Conclusion:**

This study demonstrated that site-workers and villagers had different malaria prevention behaviours and care-seeking practices even though they resided in the same geographic area. Hence, it is important to recognize such differences for more effective intervention approaches.

## Background

Globally, malaria morbidity and mortality declined between 2010 and 2016 [1]. However, it is still one of the important public health issues in the Greater Mekong Subregion (GMS) including Myanmar, Cambodia, the Lao People’s Democratic Republic (PDR), Thailand, Vietnam, and Yunnan Province of the People’s Republic of China with the emergence of a resistance to artemisinin-based combination therapy [2]. Among GMS countries, Myanmar bore the heaviest malaria burden in 2016 [3], in spite of malaria case reduction by 82% and death reduction by 93% between 2012 and 2017 [4]. Within Myanmar, northern regions near China experienced a high malaria burden and frequent outbreaks [5].

Regarding the risk factors of malaria, there are forest-related activities, difficulty in implementing and strengthening control interventions, remoteness, presence of exophagic and exophilic vectors, poor lifestyle, poor education standards, and low risk perception in GMS countries [6]. Among those risk factors, the study in Vietnam pointed out forest-related activities as an important factor with a population attributable fraction of 53% [6]. In Northern Myanmar along the border with China, which has been neglected for malaria control, the thickly forested geography, difficulty with access to infrastructure, sustained cross-border access, and political conflicts cause major barriers to malaria control, facilitating transmission to nearby regions [5]. In addition, the lack of physical, monetary and human resources leads to malaria outbreaks in these areas [5].

Due to the changing economy, the migrant worker population is rising in the GMS [7]. Mobile and migrant workers are more likely to be exposed to malaria and act as carriers of drug-resistant malaria [2]. Their nature of work and seasonal migration encourage disease spread from endemic areas to malaria-free ones in the GMS [7]. Despite the fall in morbidity and mortality in recent years, Myanmar has been experiencing a burden of drug-resistant malaria, especially on the Thailand-Myanmar border, more likely due to population mobility [8]. In Myanmar, as mobile migrant workers tend to move frequently, bringing along their families for their living and/or job availability, this prevents them from getting sufficient health information and accessing quality health services and increases their likelihood of receiving low-quality drugs, delayed diagnosis, poor treatment and inadequate follow-up [2]. These factors hamper malaria elimination efforts and contribute to the emergence of drug-resistant malaria [2].

Regarding treatment-seeking behaviour, socio-economic factors, health knowledge, beliefs, and access to health services influence patients’ health-seeking behaviour [9]. A study in Lao PDR and Cambodia showed that various socio-economic factors and/or culture and belief of the specific mobile group play a role in acceptance and compliance to antimalarial treatment [10]. People begin doing self-treatment or receiving treatment from traditional healers and then, seek care from private drug sellers, with few going to public facilities directly [11, 12]. In a household survey in Cambodia, the percentage of people who sought healthcare from private providers was two-thirds [13].

In Myanmar, a considerably large number of the population seek treatment from the private sector, including non-formal providers [14]. A survey in Myanmar showed that almost 50% of people sought medical attention from private providers, who were more likely to administer anti-malarials without any diagnostic test [15]. Moreover, the study in Wa township showed that approximately 80% of patients with febrile illness sought care from the retail sector (drug stores, shops and market stalls) [16].

Therefore, it is important to understand the role of different health providers and engage with them appropriately to facilitate malaria elimination goals [9]. The informal providers located in remote areas have a tendency to provide healthcare for poor and marginalized populations [17]. In addition, mobile and migrant populations are less likely to receive healthcare from formal health services [18]. Although mobile and migrant populations encountered fewer barriers while getting treated by informal providers, the health services and treatment received were of sub-standard quality [7].

With funding from the Global Fund, Population Services International (PSI) Myanmar planned to expand its community health service provider network through non-formal providers to be able to serve mobile and migrant populations better in four high burden and conflict-affected townships (Hsipaw, Kutkai, Kyaukme and Lashio) in Nothern Shan state near the Myanmar-China border. Along the border, there were high malaria prevalence and weak malaria services in health facilities due to lack of experienced health workers and medical equipment [19]. In addition, its geographical presentation of hilly and heavily occupied forests and increasing numbers of mobile and migrant occupants also favor malaria transmission [20–23]. Prior to implementing PSI Myanmar’s project, this study was conducted to be able to learn malaria prevention behaviours and treatment-seeking behaviours of villagers and mobile and migrant workers who resided in the same geographic area.

## Methods

### Study design

A mixed method approach was applied to explore the malaria preventive measures, health-seeking behaviours and barriers to access health care among mobile, migrant, and general populations working or residing in malaria-at-risk areas of four townships in Northern Shan State (namely, Hsipaw, Kutkai, Kyaukme and Lashio). In this study, *mobile* is defined as those persons present in the study location for less than 6 months, while *migrant* means persons who move between townships and are present in the study location for more than 6 months, but less than 12 months [24]. “Ethnic minority” persons are those from ethnic minority groups as determined by the 2014 Myanmar Housing and Population Census, which are not 8 major national ethnicities [15]. During August and December 2016, PSI Myanmar listed the number of worksites located in malaria-at-risk areas in these townships using a proper census approach. That list included the number of worksites and their worker size. Prior to this study, that worksite list was reviewed and updated after conducting a group work mapping exercise based on perceived knowledge of the basic health staff assigned to the study areas. This exercise was conducted at the respective township medical offices in early March 2019 with the aid of the Township medical officers and Township Vector Borne Disease Control team. Then, the worksites on the list and the villages located the nearest to these listed worksites were approached, and one of the representatives was each interviewed using both a structured quantitative questionnaire and a qualitative interview guide. The quantitative structured questionnaire aimed to explore the size of mobile and migrant populations, their mobility, and their malaria preventive measures in both worksites and villages. The qualitative questions explored health-seeking behaviour, provider preference, and barriers to health care among those populations.

### Study population

The study population was the key stakeholders of these worksites; worksite managers, and owners, who were approached for interviews. Additionally, the nearest villages to these worksites were identified, and each village leader or representative was asked for interviews. Finally, 23 worksites and 20 villages were approached for quantitative interviews. Of those approached worksites and villages, 14 qualitative interviews each were conducted.

### Data collection

Field data collection was conducted by PSI Myanmar research team in March 2019. Electronic data collection using the Census and Survey Processing System (CsPro) software version 7.1 was applied. The quantitative questionnaire included questions related to the basic information of businesses and villages and malaria preventive measures among those populations. For the qualitative in-depth interviews, the PSI Myanmar research team prepared a draft interview guide, which mainly included questions about health-seeking practices for any febrile illness and malaria-suggestive symptoms. The question guide was subsequently improved after conducting a pretest with the PSI’s experienced researchers. The guide included prompts that elicited storytelling from interviewees, allowing participants to provide information at their own pace and using probes to elicit additional detail. The qualitative interviews were audio-recorded.

### Data analysis

Quantitative data analysis was conducted in STATA 14.2 (© StataCorp, College Station, TX) [25]. The population size of mobile and migrant workers and villagers was calculated based on the responses to the separate questions on each group of mobile and migrant population working in the interviewed worksites or residing in the approached villages. Additionally, their common malaria preventive practices were explored and compared between the worksites and the nearby villages using the Pearson’s Chi-squared test. A statistical test with a p-value less than 0.05 was assumed to be significant.

For the qualitative data, the audio recordings were first transcribed into the local language, Burmese. Then, an initial code frame was developed based on transcripts and semi-structured questionnaires. The transcripts were coded and the code frame was updated throughout the coding process to account for any emerging themes. After that, the codes were grouped into code families and checked to see whether they gave rise to patterns of information. A chart was designed by grouping the codes into code families, categories and themes and verified by the analysis team. This procedure was carried out for each of the transcripts. Subsequently, the themes under the topics of treatment-seeking practices and barriers were developed using a thematic approach in analyzing the contents of interviews. Finally, 8 determinants for treatment-seeking behaviours and 4 types of barriers were established based on importance and patterns of information to meet the study objectives. Then, they were reported by frequency of the topic. Data coding was conducted at Atlas. ti software version 7.1.7, which is a powerful workbench for qualitative analysis by arranging, reassembling, and managing qualitative data of text documents, photos, audio clips, or video types in creative and systematic ways [26].

### Ethical considerations

This study has been approved by the Ethics Review Committee on Medical Research Involving Human Subjects, Department of Medical Research, Ministry of Health and Sports, Myanmar (Ethics/DMR/2019/007) and the PSI Research Ethics Board (#21.2018).

## Results

### Population size estimates of general, mobile, and migrant people

There were 1327 workers employed in the approached 23 worksites, with a median of 33 workers per worksite. Among those workers, approximately 34% were women with 2% of pregnant women and 7.9% were children aged under five. In the 20 nearby villages, there were 10,352 villagers with a median of 424 villagers each. More than half of the people residing in the villages were female, with 2.67% of pregnant women and 13.3% of the villages’ population being under five children.

Overall, a higher number of mobile and migrant people resided in the worksites than in the nearby villages (p < 0.001). Representatives of 20 worksites reported that they had migrant workers, who had moved to their workplace at any time since their birth, whereas 12 village heads reported so. 14 out of 23 worksites had mobile populations and 11 had migrants. Of the 20 interviewed villages, only 3 and 4 villages had mobile and migrant residents in their villages, respectively. In addition, the percentage of any mobile migrant population since their birth was higher in the approached worksites than in the approached villages (66.3% vs. 6.0%). The percentage of mobile population living in the study locations for less than 6 months was 13.0% in the worksites and 1.2% in the villages, whereas the migrants residing in the study areas for more than 6 months and less than 12 months made up of 21.3% workers for the worksites and 0.9% villagers for the villages (Table 1).

**Table 1:** Population sizes of general, mobile and migrant people in the interviewed worksites and villages

Defined indicators	Worksites	Villages nearby	P-value*
General population			
Total number of worksites/villages interviewed	23	20	
Median number of workers/villagers (min—max)	33 (6–384)	424 (35–2982)	
Total number of workers/villagers	1327	10,352	
% of female populations in the interviewed worksite/village	451 (34.0%)	5612 (54.2%)	< 0.001
% of pregnant women in the interviewed worksite/village	9^α^ (2.0%)	150^µ^ (2.67%)	0.387
% of under 5 children in the interviewed worksite/village	105 (7.9%)	1380 (13.3%)	< 0.001
Mobile and migrant population			
% of worksite/village with any mobile migrant population since their birth	20 (87.0%)	12 (60.0%)	0.043
% of any mobile migrant population since their birth in the approached worksite/village	880 (66.3%)	621 (6.0%)	< 0.001
% of mobile population^1^ in the approached worksite/village	173 (13.0%)	124 (1.20%)	< 0.001
% of migrants^2^ in the approached worksite/village	283 (21.3%)	93 (0.90%)	< 0.001

^α^N = 451, ^µ^N = 5612

*Pearson’s Chi-squared test

1 “Mobile” are those persons present in the study location for less than 6 months

2 “Migrant” mean persons who move between townships and are present in the study location for more than 6 months and less than 12 months

### Characteristics of study participants and study areas

A total of 43 participants comprising 23 worksite representatives and 20 village representatives took part in the quantitative study, whereas 14 representatives participated in the qualitative in-depth interviews. Most respondents were male for both, with a median age of 42 years for the worksite representatives and 49 years for the village representatives. All participants had at least primary education, and 8 worksite respondents graduated. Most businesses were found to be agriculture and mining for both worksites and villages nearby. Of those 23 approached worksites, 17 worksites were located within the forest while 6 were within 5 miles of the forest. Of the 20 villages nearby, 12 villages stood within the forest, while 6 and 2 villages were within 5 miles and more than 5 miles from the forest, respectively (Table 2).

**Table 2:** Characteristics of the study participants and study areas

Variables	Category	Worksites (N = 23)	Villages nearby (N = 20)
Gender	Male	20	18
	Female	3	2
Age	Median (Min–Max)	42 (25–66)	49 (25—67)
Education	Graduated	8	1
	High school standard	4	6
	Middle school standard	8	7
	Primary school standard	3	6
Townships	Hsipaw	9	7
	Kutkai	4	4
	Kyaukme	6	5
	Lashio	4	4
Position at worksite or village	Village leader	-	14
	Village representatives	-	6
	Owner	5	-
	Manager/Supervisor	14	-
	Worksite representatives	4	-
Types of worksites/Current main business of villages	Agriculture	10	8
	Factory	3	4
	Farming	2	2
	Mining	6	4
	Construction	2	2
Location of worksites/villages	Within the forest	17	12
	Within 5 miles from the forest	6	6
	> 5 miles from the forest	0	2
Types of studies conducted	Only qualitative in-depth interviews	14	14
	Both structured quantitative and qualitative interviews	23	20

### Internal migrations into the study areas

**Fig. 1:**
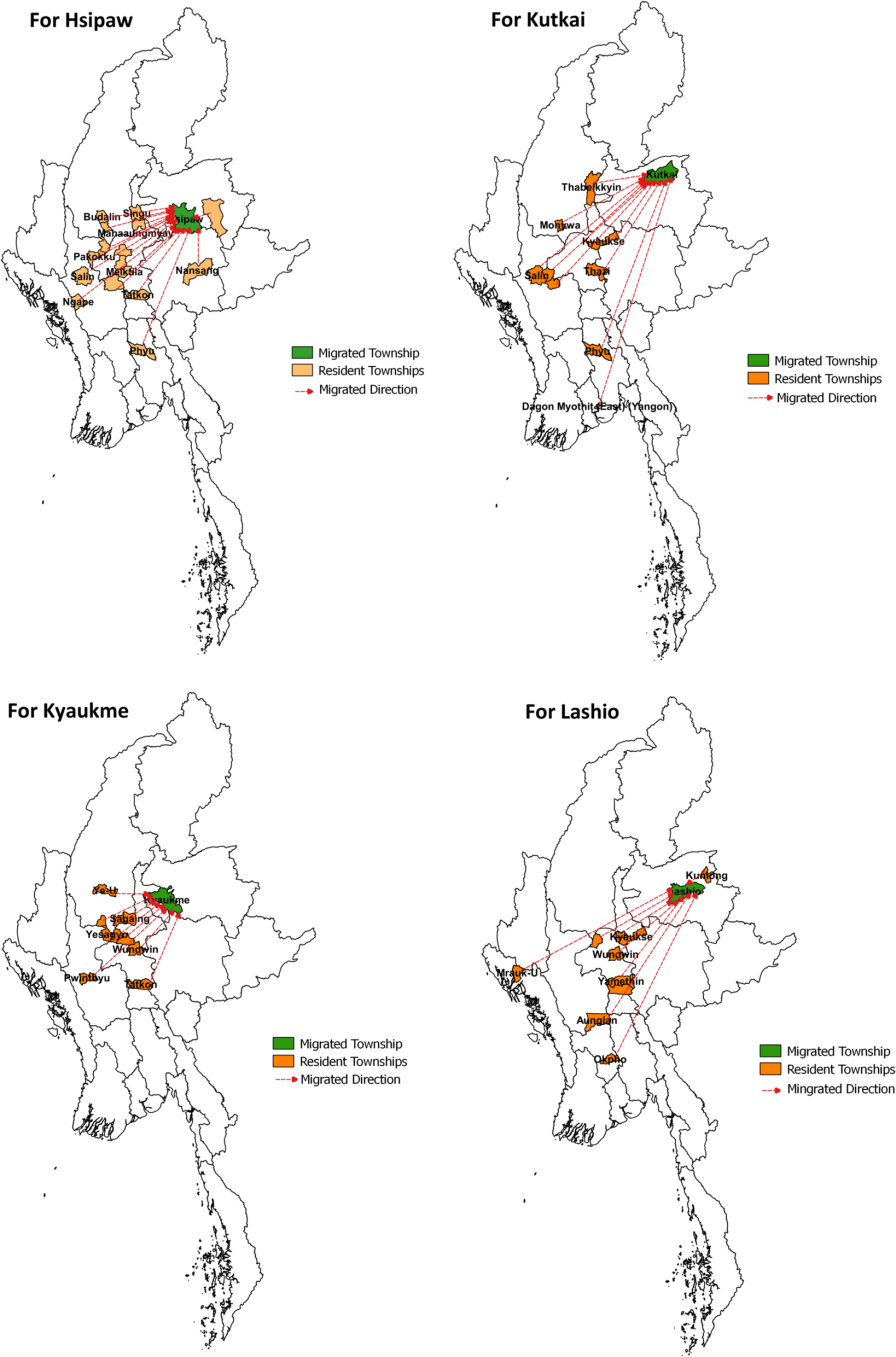
Maps showing the internal migrations from resident townships to migrated townships

The worksite representatives and village heads reported that the majority of migrants moved from the central regions of the country, mainly from Magway, Sagaing and Mandalay Divisions. In addition, some migrants working in Lashio worksites moved from Mrauk-U, Rakhine State, and intra-state migration was also noted in Hsipaw Township (Fig. 1).

### Malaria preventive measures

Regarding the questions on practices of malaria preventive measures, 73.9% of worksite managers and 85% of village heads responded that their workers or villagers usually slept under bed nets, whereas 39.1% of the worksites and 80% of the villages mentioned ITN/LLIN use (p = 0.007). Secondly, 30.4% of the worksites and 65% of the villages, respectively, had a practice of burning incense or coils for the purpose of mitigating mosquito bites. Moreover, 8.7% of the worksites used to burn leaves, aiming to eliminate mosquito reservoirs and repel mosquitoes, while only 4.35% used insect repellents. For villages, 30% of the respondents answered that they usually burned leaves, whereas 15% applied insect repellents to mitigate mosquito bites. Moreover, 47.8% of the worksites and 75% of the villages responded positively to the practice of their residents wearing protective clothing to prevent them from mosquito bites. However, taking malaria prophylactic drugs was no longer a popular choice, making up only 4.35% of the worksites and 10% of the villages adopting such a practice (Tables 3, 4 and 5).

**Table 3:** Malaria preventive measures

Defined Indicators	Worksites (N = 23)	Villages nearby (N = 20)	P-value*
Sleeping under bed net			
Yes	17 (73.9%)	17 (85.0%)	0.373
No	6 (26.1%)	3 (15.0%)	
Sleeping under ITN/LLIN			
Yes	9 (39.1%)	16 (80%)	0.007
No	14 (60.9%)	4 (20.0%)	
Wearing long clothes, gloves or using a scarf			
Yes	11 (47.8%)	15 (75.0%)	0.069
No	12 (52.2%)	5 (25.0%)	
Burning incense or a coil			
Yes	7 (30.4%)	13 (65.0%)	0.023
No	16 (69.6%)	7 (35.0%)	
Burning leaves			
Yes	2 (8.7%)	6 (30.0%)	0.073
No	21 (91.3%)	14 (70.0%)	
Using insect repellent on the skin			
Yes	1 (4.3%)	3 (15.0%)	0.23
No	22 (95.7%)	17 (85.0%)	
Taking medicine to prevent from malaria			
Yes	1 (4.3%)	2 (10.0%)	0.468
No	22 (95.7%)	18 (90.0%)	

*ITN* Insecticide-treated net, *LLIN* Long lasting insecticidal net; *Pearson’s Chi-squared test

**Table 4:** Factors determining health-seeking preferences among worksite workers and villagers

Descriptions	Similarities	Differences
Local and traditional belief		
Local belief that taking traditional/local medicine and herbs and doing traditional practices (e.g., “*Makalaung*”—an action of piercing fingertips and toe tips with needle) would certainly relieve fever prior to or together with western medicine. Some also seek treatment from shaman due to spiritual and traditional belief	Traditional belief and local routine health practices influenced both residents and migrants to take such traditional means as the most commonly reported initial treatment option	These methods were quite more common among villagers. Despite these methods not being popular among migrants at first, the locals persuaded them to espouse such practices from them
Easy availability		
Easy availability of traditional or western medicine at home or nearby drug stores encouraged self-medication and made convenient to take medicine on their own. Self-treatment involved taking medicine themselves or cocktail drugs purchased from nearby drug stores. The medicine they most often used were analgesics (such as Decolgen, paracetamol, Neomixagrip), traditional drugs (e.g., Shan drug power) and cocktail unspecified drugs	Self-treatment served as the popular choice for both villagers and worksite workers as one of the initial treatment options	No difference
Arrangement by worksites		
Providing a medical box with western and traditional medicine for symptomatic relief at most of the worksites promoted workers to choose as the initial treatment Some worksites planned for their workers to seek health services at social welfare health centres without any charges Also, some worksite owners arranged transportation for their staff to seek health care at the formal health care services	No similarity	All these mentioned services only available at some worksites. From a medical box, the staff who administered drugs did not have any medical-related certificates No medical box available at village
Local recommendation		
The word-of-mouth recommendation by neighbours determined choice on health service providers, which may be either informal providers, basic health staff, medical doctors or community health providers	In some instances, workers and villagers directly seek health care at health service providers without taking any prior self-medication	No difference
Service cost		
Low cost to purchase medicine on their own Received treatment from informal providers and basic health staff by credit payment, which means that patients could pay service charges later, by the time patients could afford or after seasonal harvesting period	Low cost to buy medicine at drug store encouraged self-medication among all locals and workers rather than seeking care at health providers	Credit payment was more common among villagers despite being available for both
Accessibility		
Nearby health service providers or drug stores Home health services by informal providers and basic health staff Accessible to malaria testing and treatment services via community health service volunteers	Nearby drug stores and home service by informal providers and basic health staff gave an access to health services among locals and migrants	Most migrants sought care from basic health staff nearby Some villages had the trained community volunteers who could provide malaria services to villagers, but very few worksites’ representatives reported volunteer services
Trust and relationship		
Quality of services, prior good relationships and friendly nature of health services providers influenced care receivers to choose one of health care providers (either basic health staff or informal providers or medical doctors) Trust on the medical proficiency was one of the determinants in selecting formal health care providers No communication barriers	All the mentioned reasons were important for both locals and migrant workers in selecting health service providers Both migrants and locals preferred formal health care by medical doctors and basic health staff due to trust on medical proficiency	Migrant workers chose formal health service providers (medical doctors or basic health staff) who had no language barriers with them whereas locals favored informal providers and local health staff owing to being friendly and able to speak local Shan languages
Disease severity		
Seeking health care services at the formal health centres and/or public hospitals happened at the conditions when symptoms became worsen and severe symptoms of disease not cured by initial treatment	Mostly, both locals and migrants chose the formal health centres or hospitals for their secondary source of health care	No difference

**Table 5:** Barriers in seeking health care

Barriers	Descriptions	
	Worksites	Villages
Health facility barriers		
Inaccessible to formal health service facilities	Insufficient numbers of government health staff and worksite locating in hard-to-reach area	Insufficient numbers of government health staff
Unavailability of diagnostic test	No experience of getting diagnostic services	Same as mentioned in worksites
Poor communication by health staff	No language barriers with government health staff	Local residents have language barriers in communicating with Burmese health staff, which often leads to misunderstanding between health providers and patients
Self-barriers		
Fear and trust	Fear of visiting health service providers and prior no experience at health care facility, and prior negative experience causes them to delay seeking care at formal health facility	Fear of visiting health service providers, language barriers, no prior experience at health care facility, and prior negative experience caused them to delay seeking care at formal health facility Moreover, villagers did not have trust on community volunteers trained by government and /or non-governmental organizations
Socio-economic barriers		
Financial difficulties and work nature	Mostly were seasonal workers and daily-based ones, with no spare money to be able to afford both transportation and medical charges at formal health care facilities. Daily-based workers were often reluctant to take a leave from work for their income, so mostly relied on self-medication or nearby drug shop	Same as mentioned in worksites
Limited health knowledge	Limited access to health education activities and negative perceptions towards health education talks	Same as mentioned in worksites
Location barriers		
Hard-to-reach area	Mostly located in hard-to-reach area, devastated road condition and often needed multiple modes of transport to reach health centres	Same as mentioned in worksites
Insecurity by frequent armed conflicts	Insecure on the way to health facility Restricted workers not to go beyond the worksite compound due to frequent armed conflicts and worksite nature	Insecure on the way to health facility Village authorities must ask permission from armed conflict groups to use the way to health facility

### Factors determining health-seeking preferences

Face-to-face interviews with 14 worksite representatives and 14 village representatives revealed that there were 8 determinants which influenced both worksite workers and villagers in seeking health care when they had fever: (1). local and traditional belief; (2). easy availability; (3). arrangement by worksites; (4). local recommendation; (5). service cost; (6). accessibility; (7). trust and relationship; and (8). disease severity.

### Local and traditional belief

Treatment by traditional means was the most reported initial treatment option for both types of respondents. Treatment by traditional means included taking traditional or local medicine and herbs by themselves or recommended by their coworkers or villagers and doing traditional practices called Makalaung, an action of piercing fingertips and toe tips with a needle, and/or Sar-chit, which is an action of scratching the skin with a sharp object until the skin is red. They strongly believed that such activities could relieve fever. In addition, some sought treatment from shamans due to their spiritual and traditional beliefs. These traditional methods were quite common among villagers due to local beliefs. Although the migrant/mobile workers did not have such practices initially, they espoused such practices from residents and later tried to practice these methods by themselves or took assistance from residents whenever they had a fever. In that way, traditional practices became commonly used among those communities, and they supposed that the medicine and injections they took would be effective only after receiving such traditional practices.

One village representative answered:

“The skin has to be scrapped with a tin of condensed milk after applying pain-relieving ointment. Then, the skin becomes red. It is like opening sweat pores. After that, we take analgesics. Some people take traditional antipyretic medications. We sleep after taking these drugs. When we wake up, we feel lethargic, but our symptoms are relieved. There is a traditional belief like that.” (Village Head from a village in Kyaukme 05).

One village head explained:

“The people who come from Myanmar (plain area) do not believe Makalaung. However, later, they sought treatment from local health providers and received Makalaung.” (Village Representative from a village in Kyaukme 02).

### Easy availability

Self-medication also served as the popular choice for both villagers and worksite workers owing to the easy availability of western and/or traditional medicine at home or nearby drug stores. Self-treatment involved taking medicine themselves or cocktail drugs purchased from nearby drug shops or stores. The medicine they most often used were analgesics (such as Decolgen (Chlorpheniramine maleate), paracetamol and Neomixagrip (Paracetamol and Chlorpheniramine maleate)), cocktail unspecified drugs, and anti-malarial medicine, commonly referred to as “Artesunate”. It was also reported that very few people who had a history of malaria-like febrile illness took anti-malarial drugs at the time of their febrile illness without any malaria diagnostic tests.

One village head mentioned:

“If I sell drugs here, they buy one tablet (analgesic) for 50 or 100 Myanmar Kyats. The symptoms of some people relieve themselves in this way. Some people who do not get well after the medication go to clinics in the city.” (Village Head from a village in Hsipaw 01).

One worksite representative expressed:

“There are some drugs they (the workers) are used to taking. For example, we take paracetamol if we have a headache or Biogesic if we have a cold. Like that.” (Owner of an agricultural worksite in Hsipaw 01).

### Arrangement by worksites

In most of the worksites, a medical box with western and traditional medicine for symptomatic relief was provided, which was found to be initially and widely used by worksite workers as an initial source of treatment. Some worksites even appointed staff who were able to administer medicine. However, some staff who administered drugs did not have any medical-related training or certificates. They usually dispensed drugs with their own general knowledge.

One worksite representative shared his experience:

“Mild diseases are headache and fever. We don’t usually suffer from fever. If they suffer from loose motion, I administer them metronidazole. I tell them to take drugs based on my knowledge.” (Manager of an agricultural worksite in Kyaukme 04).

A few worksite representatives claimed that their worksite workers sought treatment at a social welfare clinic. That clinic offered free-of-charge treatment because of the arrangement by the worksites. However, one worksite representative reported that free-of-charge services at social welfare health facilities could be available to current workers, but not to their family members.

The worksite representative stated:

“Only people who have employee identity cards seek treatment from places which offer free-of-charge services.” (Manager from a farming worksite in Kyaukme 03).

Moreover, as an additional service by worksites, there was a transportation arrangement to seek health care at a social welfare health facility, the nearest rural health centre, or private clinic.

“At the moment, as our workers have motorcycles, they go to health providers even if they suffer from nausea or headaches. If they cannot walk there, we send them by our worksite car. Most importantly, because of the easy transportation, they can easily reach a health centre” (Manager from a mining worksite in Lashio 01).

### Local recommendation

Both worksite and village interviewees mentioned that the workers and villagers in some instances directly sought health care from health service providers, such as basic health staff, informal providers, and medical doctors. The word-of-mouth recommendation by neighbours/coworkers determined their choice of health service providers.

“When a person in a village provides treatment for an illness, the locals tell others to go there for treatment. They end up receiving treatment there like that.” (Village Representative from a village in Kyaukme 02).

### Service cost

Both villagers and migrant workers could purchase western or traditional medicine at a low cost in nearby drug stores, encouraging self-medication and making it convenient to take medicine on their own. Hence, they would choose this option first rather than seeking care from health care providers. In addition, the respondents reported that they could receive health care from health care providers by credit or flexible payment, which meant that patients could pay service charges later, when patients could afford it or after the seasonal harvesting period. Despite credit payments being available for both types of service receivers, self-medication was more common among villagers.

“If we tell her (the informal health provider) that I don’t have money now, she just gives us injections with credit payments. Based on a disease condition, she comes to give an injection the next day at the time when we need an injection. Like that. The cost of the treatment can be paid when the patient gets cured and can work. That has become the traditional behaviour of mountainous areas.” (Village Head from a village in Kyaukme 05).

### Accessibility

The preference of providers (basic health staff or informal providers) depended on accessibility to nearby service providers and home services provided. It was often reported that most migrants sought care from basic health staff nearby, whereas villagers chose a private clinic if their vicinity was closed to the town, as most private clinics were located in the town. Moreover, the village representatives reported that there was a community-based health service provider who could provide rapid malaria diagnostic tests, anti-malarial medicine, and referral service to health centres if a patient did not relieve symptoms after his treatment. However, the representative of one worksite reported such volunteer services.

“It is convenient to seek treatment from a village midwife because we don’t have to go to her place. She comes to our houses and gives us treatment.” (Manager from a farming worksite in Kyaukme 03).

“The informal providers give home visits when they are asked to provide home service. They don’t care about financial issues. They just help if they are asked for home visits.” (Village Representative from a village in Kyaukme 02).

“He (a malaria volunteer) provides blood test services. He takes blood from his fingertips and gives him medication. They are all free of charge.” (Village Representative from a village in Hsipaw 04).

### Trust and relationship

Mutual trust, good prior relationships, and the friendly nature of health service providers influenced care receivers to choose one of the health care providers (either basic health staff or informal providers or medical doctors). The worksite representatives reported that there was no language barrier between workers and the basic health staff because most of the government staff could speak Burmese well. In addition, workers much more preferred basic health staff than informal providers. However, traditional healers or informal providers were more popular among the villagers owing to trust, being friendly, belief in traditional ways of treatment and being able to speak local Shan languages.

A worksite representative praised how the health assistant at her village could cure any diseases.

“We have a health assistant here. Moreover, there are 3–4 nurses. The medical proficiency of the health assistant is good. They can cure mild cases. We don’t have to go anywhere. We don’t even need to go to the city.” (Owner of an agricultural worksite in Hsipaw 01).

One villager shared his experience of the language barrier:

“I think that the health assistant is from Monywa. Thus, he doesn’t know how to speak in the Shan language. Most importantly, language. If a person does not know how to speak in the local language, it is a little difficult for us to communicate with him. Thus, we rely more on a person who we can communicate freely with.” (Village Head from a village in Kyaukme 05).

On the other hand, one worksite representative shared different stories of language barriers:

“As health providers communicate in the local language, we do not have any other choice. As we are Burmese, we are afraid that the treatment given by these providers is not appropriate for us. Thus, we sought treatment from Sayarmalay’s clinic, which is a Burmese clinic. We dare not seek treatment from local clinics.” (Owner of a construction worksite in Hsipaw 05).

In addition, quality of health services, trust in the medical proficiency and knowledge of medical doctors were the reasons why formal private doctors and basic health staff were approached as both initial and secondary ways of treatment. Moreover, previous good memories of quick recovery and severe disease conditions also encouraged them to prefer formal health care providers.

The village head and worksite representatives shared their views on the preference of private doctors.

“They (the villagers) go to places where there are doctors. They get examined by doctors. The doctors use stethoscopes. Thus, they know the conditions of the patients (both inside and outside). Therefore, people mostly say that it is more certain to go to places where doctors give treatment.” (Village Head from a village in Hsipaw 01).

“As the workers come from the central part of Myanmar, they don’t believe anyone apart from doctors. Thus, they don’t seek treatment from any other provider.” (Manager from a mining worksite in Lashio 01).

### Disease severity

If a patient was not recovered by any initial treatment (mostly traditional methods and self-medication), they usually sought secondary sources of health care at rural health centres, private clinics, or public hospitals. Basic health staff and private clinics were the most often reported ways after initial self-treatment by either traditional or western means. The reasons for preferring such basic health staff and private clinics were the same as mentioned above, such as trust in quality of services, home visits, friendly nature of staff and flexible payment.

One village head explained:

“If we suffer from a headache or cold, we buy drugs from drug stores and take them. If the illness was not relieved after treatment, we ask Aunty……. (a midwife) to give us an injection. Like that.” (Village Head from a village in Hsipaw 02).

Similarly, one worksite representative shared their health-seeking practices as follows:

“First, we do Makalaung and then we take traditional medication. We add paracetamol for headaches. Then, we go to Sayarma (a basic health staff) to get one or two injections.” (Owner of an agricultural worksite in Kutkai 02).

The government hospital was the last option if a sick person was not cured by initial formal and non-formal health services at their place. They mentioned that they would choose a hospital only for conditions when they believed symptoms would worsen and hospitals could provide 24-h service. One worksite representative shared her thoughts:

“Sometimes, I mostly send my employees to Lashio Hospital for their benefit (if their symptoms don’t get better). I don’t usually send them to Hsipaw. There are more health providers in Lashio. In addition, it is a township. Lashio is a bigger township than Hsipaw. Therefore, it is more reliable for us.” (Owner of an agricultural worksite in Hsipaw 01).

### Barriers in seeking health care

Barriers to seeking health care reported in this study were health facility barriers, self-barriers, socio-economic barriers and location barriers.

### Health facility barriers

In terms of health facility barriers, the participants expressed inaccessibility to formal health service facilities, unavailability of diagnostic test, for example, malaria rapid diagnostic test (RDT), and poor communication by health staff in their residency, which led to delay in getting proper treatment among their workers and villagers. They experienced insufficient numbers of government health staff because only one health staff had to take responsibility for seven or eight villages and that health staff had frequent travel to the town for meetings. Some reported that they did not have any testing experience for fever cases, and most health providers usually prescribed medicine at first to relieve symptoms and/or referred very ill patients with or without malaria presumptive symptoms directly to hospital. Therefore, they thought that testing services for malaria were not available in their region.

Poor communication reportedly happened more frequently in villages than in worksites. Residents mostly could not speak Burmese language well. That made them reluctant to speak with Burmese health staff. A few village heads complained that most public health staff and hospital staff did not show their hospitality and sometimes scolded them, especially in cases of not being able to buy the necessary medicine they asked for. This often led to misunderstandings between health providers and patients. Such situations of language barriers and unwelcome behaviour of formal health staff caused them not to seek care at public health centres if the condition was not severe. However, worksite workers encountered language barriers in a different way. As most of the mobile and migrant workers were Burmese, they did not understand or speak the local Shan language. Therefore, they had trouble buying drugs or seeking treatment from local traditional healers. Thus, they usually went to health providers who could speak Burmese language well, mostly government staff.

One village head shared:

“I think that there may be some misunderstandings between some people and doctors and nurses from a hospital because these people don’t understand their questions due to language barriers. Sometimes, there may be some misunderstandings of the instructions of medical personnel. Therefore, some illnesses that can be cured within 10 days take about a week to recover.” (Village Head from a village in Hsipaw 01).

One worksite representative said:

“As this is an ethnic village, the locals mostly seek treatment from local health providers. As the local health providers are of the Shan ethnic group, most Shan get medical attention from them. As we are Burmese, we can only go to Sayarma (a basic health staff) who can explain to us (about our disease condition) properly in the Burmese language well. We all go to her if we get hurt from a minor worksite injury.” (Owner of a construction worksite in Hsipaw 05).

### Self-barriers

A few participants reported that most local residents and workers had a fear of visiting health care facilities and speaking with health service providers. This intrinsic fear caused them to delay seeking health care at health facilities and was further exacerbated by language barriers between the villagers and medical staff. Having prior negative experience at health centres also prevented both villagers and worksite workers from visiting health staff, whereas no prior experience of visiting health facilities exhibited the reluctance of local people to seek treatment at health centres. Furthermore, village heads reported that although there were community volunteers who received training from government or non-governmental organizations, most villagers did not have trust in them as they thought that they had less experience and were at a young age. Also, they complained that volunteers could provide only rapid diagnostic tests for malaria, not other diagnoses, or treatment. Therefore, people rarely visited those volunteers for their general health care.

One village head reckoned:

“As you know, the people from the mountainous regions are afraid of clinics and hospitals. Everyone fears going to them.” (Village Head from a village in Kyaukme 05).

A worksite representative said:

“People are afraid of hospitals as they have no experience of dealing with them. They fear communication. They also have more or less financial difficulties. Some people might have a fear of Sayarma (a health provider) since they have had no experience of dealing with her since their childhood and of injections.” (Manager from a farming worksite in Kyaukme 03).

“People don’t trust him (a malaria volunteer) as he is kind of inexperienced. He only provides malaria diagnostic tests to check whether a person suffers from malaria. Like that. He only provides blood tests without any treatment. That’s why people don’t go to and trust him. If people are suspected of suffering from malaria, they come to him for a blood test. Like that. I have never heard that he administers antimalarials.” (Village Representative from a village in Kyaukme 02).

### Socio-economic barriers

Both village heads and worksite representatives mentioned that villagers and worksite workers had financial difficulties accessing proper health care. Most workers relied on seasonal jobs and most residents on seasonal farms for their livings. They did not usually save extra money for their health as well as no health coverage by most of the worksite owners. In addition, their daily incomes and health care costs were almost the same, at least for one-time health care. Therefore, it was challenging for them to afford both transportation charges and medical fees to seek treatment at formal health centres or hospitals. They added that it would take time to reach health centres, so they might need to take time off work to seek health care. Most workers in both worksites and villages were daily wage earners. So, they lost their daily income if they took time off to seek health care. That was also another reason why residents and workers had to take a palliative drug at a nearby drug store or self-medication to relieve their symptoms.

“People have financial difficulty. In our villages, we earn money only at the harvesting season of corn. Only once a year! The people who own pineapple plantations earn money twice a year. The people who only plant corn earn money once a year. We only do corn plantations. Nothing else.” (Village Representative from a village in Hsipaw 04).

“As they are daily wagers, we cannot give them leave like that (for seeking health care). If they fail to work (for a day), we cannot give them their daily wage. Besides the daily-wagers, there are also permanent staff like administrative staffs. We give them leave.” (Manager from an agricultural worksite in Kyaukme 04).

Furthermore, both worksite representatives and village heads reported that most residents and workers had poor health knowledge due to limited access to health messages and talks. They usually perceived negative attitudes towards health education too.

The village head said:

“Some people think that they gain nothing from health talks as treatments are not given there. In their minds, they don’t think that they can gain much knowledge if they really listen to this knowledge and keep it in mind. Some people have a fair amount of knowledge which they should know if we really ask them.” (Village Head from a village in Hsipaw 02).

One worksite representative explained that the low health knowledge of his workers led them to do self-administration of drugs:

“I: Why do you buy drugs from outside drugstores? What difficulty do you have?

R: Some people don’t even know about their health conditions. They say that they suffer from fever with chills and rigors, but they don’t know that it is a sign of malaria.” (Owner of a construction worksite in Hsipaw 05).

### Location barriers

As the interviewed worksites and villages were mostly located near or within the forest, transportation difficulties to reach health care centres were commonly reported for those residing in hard-to-reach vicinities. They normally needed multiple modes of transport to reach the nearest health centre in their residence. In the unexpected and unpleasant weather conditions, as roads were mostly damaged, it was hard to transport patients to health centres. Even in the dry season, they could not reach health centres at night. Such situations of devastated roads in their areas still hindered them from reaching health facilities. A few village heads mentioned that insecure conditions within their residency were also one of their barriers to seeking proper treatment at a health care facility. During an armed conflict period or whenever there were armed groups on the way to a health facility, village authorities must contact both sides of armed conflict groups and ask their permission to use a road to a health facility. Even in the cases of emergency conditions that happened at night, they had to wait until morning to send patients to the nearest health facility. Therefore, insecurity was the main barrier in this community, delaying them from seeking proper health care at health facilities.

“In our village, we have only ten households. The insurgent groups come to a village regardless of the number of households. When they come to a village, the village head or administrator knows the leaders of these groups. We have their contact numbers given by these groups. If we encounter difficulties (health-related ones), the village head or administrator can make a call to them. For us who live in a hilly region, an armed conflict occurred in our village, Top-San.” (Village Head from a village in Kyaukme 05).

One worksite representative cited that worksite owners usually restricted their workers from going beyond their worksite compound, especially at nighttime, due to their nature of work and insecurity issues nearby. If workers went outside a worksite compound in their own minds, the worksite authorities could not guarantee their safety.

He said:

“R: They (workers) go outside at night. Frankly speaking, due to the current situation, nature of work and environmental conditions, we restrict them from going outside. We take responsibility for their safety if they live within the worksite compound. But I don’t allow them to go outside. If anything happens when they go outside, I am not responsible in that case.” (Manager of an agricultural worksite in Kyaukme 04).

## Discussion

In this study, a considerably larger number of worksites reported that although workers were not opposed to sleeping under bed nets, they did not use LLIN as compared to the villagers of nearby villages, despite both populations residing in the same malaria endemic area of Northern Shan State. That could be explained by insufficient LLINs in the worksites, barriers to LLIN distribution to these worksites in the study areas, and poor knowledge and bad attitudes of workers to LLIN use and its benefits against malaria [27]. Self-treatment was a popular first treatment option in both village and worksite populations. Despite the similarity, these two populations were different in that while the villagers were said to be relied on self-treatment with traditional methods and leftover drugs at home, the workers were reported to mostly take drugs from medical boxes provided by the worksites. Moreover, the villagers were said to rely more on informal providers, whereas the worksite representatives said that the workers preferred formal providers like basic health staff. Furthermore, transport arrangements and social welfare services were available only to workers. Villagers and workers alike went to government hospitals only for severe disease conditions. Insufficient manpower for formal health services, limited access to malarial diagnostic tests, communication difficulty with service providers, fear, transportation difficulty and insecure living conditions due to frequent armed conflicts were the most reported barriers in seeking health care for both workers and villagers. While poor communication with health providers happened more frequently in villagers, inability to seek care due to worksites’ location in hard-to-reach areas and transportation barriers were frequently found among workers. These findings point out the need for programs to fully understand the root causes of limited access to health care. The differences and similarities between these two populations can be leveraged to deliver effective malaria control interventions.

People in the study areas were found to have limited access to quality treatment and diagnostic tests for malaria. Most of them did not know where they could receive RDT for malaria diagnosis and most health providers gave them treatment without any tests, leading them to think that RDT testing was not available in their regions and they took medication or went directly to health providers. From this situation, it could be assumed that the usage of RDT was low in the study areas. The low level of RDT testing had been previously discovered in the study of ethnic minority residents on the China-Myanmar Border [16]. Moreover, mobile and migrant workers used ITN/LLIN relatively less than their village counterparts (39% of worksites and 80% of villages). The findings about mobile and migrant populations were consistent with previous studies in Tanitharyi Region [2] and Thailand [28]. In the study in Tanitharyi Region, only 50% of the mobile and migrant workers slept under ITN/LLIN [2]. Similarly, in Thailand, there were 25.8% of malaria-affected households in villages where the majority of rubber plantation workers lived slept under ITN/LLIN [28]. In contrast, the household study about ethnic groups in Northern Myanmar found that 73.9% of households owned ITN/LLIN [5]. The findings highlighted health inequality in both villagers and mobile and migrant workers residing in the forest-related areas of the Northern Shan State, in terms of assessing quality of malaria diagnosis and treatment, and migrant populations were more adversely affected with lesser access to effective malaria preventive measures. However, there were study methodological differences between the previous studies and this current one in the sense that the former were household surveys while the latter did interviews with representatives of villages and worksites.

The possible explanations for these unequal malaria service accesses were unsafe and unsecure residency for both villagers and mobile migrants. Frequent armed conflicts in Northern Shan State made villagers and workers difficult to go to formal health workers in accessing required health services [29]. In addition, almost all mobile migrant workers and villagers were daily-wage earners, who could be unpaid for their absent days. This might be another reason why they were reluctant to go to formal health providers after taking unpaid leave. The worksites had more barriers to accessing health services and could hardly be reached by health workers because of the legal status of the worksites and the nature of work. It was reported that there might be either legal and illegal worksites or workers in border areas, and such illegal status hampered them from connecting with formal health networks due to fear of punishment [30]. Moreover, the lack of community health workers in most worksites consequently prevents workers from receiving timely malaria diagnosis and treatment. Hence, the National Malaria Control Programme (NMCP) and malaria partners should take account of these aforementioned possible reasons for unequal malaria access among mobile and migrant workers and village populations, and pay special attention to ITN/LLIN distribution among mobile migrant populations.

The other reason for not accessing proper malaria care was self-treatment, which was the mostly used treatment option among villagers and mobile and migrant workers. The former relied more on treating themselves with traditional methods and leftover drugs at home, while the latter used medication from medical boxes provided by their worksites. Both relied on drugs bought from drugstores near their homes. The action of villagers’ self-treatment by taking ‘left-over’ drugs at home or drugs from drugstores as initial treatment were reported in many previous studies [11, 31–34]. The reasons behind both the villagers and worksite workers practicing the self-medication were due to low cost, easy drug availability, unwillingness to take leave from work, and mild disease severity. Long travel distances to health providers and unsafe environmental conditions also added to their reluctance to go to formal health facilities. A previous study in Bago Region found that mobile and migrant workers could easily access medication at small shops or drugstores and thus, did self-treatment with these drugs [7].

In addition to self-treatment, the villagers mostly relied on informal providers and sometimes, traditional healers, while the workers preferred formal providers like basic health staff. They went to these providers either directly or after doing self-treatment or traditional methods at home. The language barrier was the commonly reported reason behind both of their preferences. Both villagers and workers preferred going to health providers who could communicate well in their languages; Shan and Burmese languages, respectively. Other reasons behind the villagers and workers’ choice of informal providers and traditional healers were home service availability, easy accessibility, having trust and good relationship with the providers, word-of-mouth recommendation by neighbours and credit or flexible payment, while both sought treatment from basic health staff for the same reasons. Moreover, the worksite workers received treatment from basic health staff because of transportation arranged by the worksites. Similarly, a previous study in the Bago Region found that easy accessibility, trust and good relations with providers caused migrant workers to seek health care from the informal sector [7].

NMCP concentrates on expanding community-based malaria case management with the help of community health workers trained by the government and non-governmental organizations [30, 35]. It found that malaria case finding through community workers in Myanmar was the most efficient approach with minimal resources required [36]. However, this study pointed out the health coverage gap in this key vulnerable population by community health workers. Although there were community health workers in some villages, most worksites did not have these providers for timely malaria diagnostic testing and treatment. From the words of some village heads, the villagers had less trust in community health workers and did not rely on them because these providers were relatively young compared to other providers and could provide only malaria diagnostic tests and treatment. However, some worksite representatives requested to send community health workers for malaria services and to train one of their workers to be ready for malaria case finding and early treatment. Therefore, NMCP and partner organizations should consider recruiting community-trusted volunteers at worksites or within the vicinity of worksites, and equipping them with malaria comorbidity and skills.

With regards to barriers in seeking treatment, both worksite workers and villagers were unable to access formal health services and diagnostic tests, and experienced communication gaps with health providers. Being daily wagers, both had financial difficulties and were reluctant to take leave to receive health care for their illness. Moreover, they both had limited health knowledge, fear, or trust problems about visiting formal health facilities. Being residing in hard-to-reach areas with poor transportation and insecure conditions, and frequent armed conflicts have worsened the situation for seeking treatment for malaria. This study is consistent with recent local studies conducted among mobile migrant workers in malaria elimination areas and among the Wa ethnic minority in Myanmar [2, 7, 16]. They reported that the remoteness of their worksites, far distance from a health facility, transportation constraints, security concerns, unsuitable open hours of clinics, and social distance hindered them from accessing formal health services [2, 7, 16]. The language barrier was also a non-negligible issue in seeking health care as the majority of the migrant population understood the Myanmar language while the local villagers speak their ethnic language, which was proved to impede their understanding of health messages from their providers [2, 10]. In addition to these limitations, financial income and lack of ready cash were also the reported barriers associated with treatment seeking in both rural ethnic Myanmar [16] and Cambodia [37]. Hence, this study was able to document a wide range of individual and contextual constraints among both mobile migrants and rural ethnic populations, which might hamper their health-seeking practices in formal health care and are likely to increase the potential risk of malaria transmission in these malaria at-risk areas. Therefore, local health authorities and non-profit health organizations should seek to increase trust and overcome geographic and language barriers that hinder access to health services in order to achieve the National Malaria Elimination targets in Myanmar [30].

This study is limited to inferences drawn from quantitative and qualitative insights across a small number of respondents in the targeted hard-to-reach and malaria at-risk areas, so that it cannot be nationally representative. In addition, since discussions were made only among key stakeholders of the study worksites and villages, not individual workers and villagers, the findings were not able to generalize to individual levels, and there might be other individual-level factors or barriers that may not be observed in this study. However, key informant interviews have several advantages to collect needed information, ideas, and insights that cannot be obtained with other methods, since the data comes directly from more knowledgeable people, who may offer confidential information that would not be revealed in other settings. They may also tell of issues, local happenings, or conditions that explain overall problems. In addition, the draft interview guide was subsequently improved after each interview to explore new ideas and issues that had not been anticipated in planning this study but that were relevant to the study purpose. As an additional strength, it was the first study that could highlight the considerable differences and gaps in malaria preventive measures and health-seeking behaviours between site workers and residents despite living in the same geographical areas.

## Conclusion

The National Malaria Control Programme, Myanmar, aims to ensure equitable and universal access to effective preventive and curative measures for all ‘malaria at-risk populations’, given a priority to the most vulnerable and hard-to-reach mobile and ethnic populations living or spending time in remote forested areas [30]. However, this study highlights the gaps in access to LLIN and malaria diagnosis and treatment among mobile migrant populations and ethnic minority people residing in Northern Shan state, which is the malaria-at-risk area of Eastern Myanmar close to the Chinese border. Low LLIN use was reported among the worksites than among the villages in the same geographical regions. Although self-medication was the primary preference for seeking care related to any febrile illness for both communities, the two study groups had different motivations for these care-seeking practices. Programmes which seek to implement malaria control interventions and facilitate malaria elimination need to recognize such similarities and differences, to strengthen and effectively design target malaria intervention approaches in those malaria-at-risk areas.

## Data Availability

Not applicable.

## Abbreviations

CsPro: Census and Survey Processing System
GMS: The Greater Mekong Subregion
ITN: Insecticide-treated nets
LLIN: Long-lasting insecticidal nets
NMCP: National Malaria Control Programme
PSI: Population Services International
RDT: Rapid diagnostic test

## References

[1] WHO. World Malaria Report 2017 Geneva: World Health Organization; 2017. https://www.who.int/publications/i/item/9789241565523. Accessed 19 Dec 2021.

[2] Wai KT, Kyaw MP, Oo T, Zaw P, Nyunt MH, Thida M (2014). Spatial distribution, work patterns, and perception towards malaria interventions among temporary mobile/migrant workers in artemisinin resistance containment zone. BMC Public Health.

[3] Cui L, Yan G, Sattabongkot J, Cao Y, Chen B, Chen X (2012). Malaria in the greater mekong subregion: heterogeneity and complexity. Acta Trop.

[4] WHO. Help prevent malaria Myanmar: World Health Organization; 2018. https://cdn.who.int/media/docs/default-source/searo/myanmar/help-prevent-malaria-(english).pdf?sfvrsn=7e71af10_0. Accessed 19 Dec 2021.

[5] Wang RB, Dong JQ, Xia ZG, Cai T, Zhang QF, Zhang Y (2016). Lessons on malaria control in the ethnic minority regions in Northern Myanmar along the China border, 2007–2014. Infect Dis Poverty.

[6] Bui HM, Clements AC, Nguyen QT, Nguyen MH, Le XH, Hay SI (2011). Social and environmental determinants of malaria in space and time in Viet Nam. Int J Parasitol.

[7] Win AYN, Maung TM, Wai KT, Oo T, Thi A, Tipmontree R (2017). Understanding malaria treatment-seeking preferences within the public sector amongst mobile/migrant workers in a malaria elimination scenario: a mixed-methods study. Malar J.

[8] Hlaing T, Wai KT, Oo T, Sint N, Min T, Myar S (2015). Mobility dynamics of migrant workers and their socio-behavioral parameters related to malaria in Tier II, Artemisinin Resistance Containment Zone Myanmar. BMC Public Health.

[9] Karyana M, Devine A, Kenangalem E, Burdarm L, Poespoprodjo JR, Vemuri R (2016). Treatment-seeking behaviour and associated costs for malaria in Papua. Indonesia Malar J.

[10] Workshop to Consolidate Lessons Learned on BCC and mobile migrant populations in the strategy to contain artemisinin resistant malaria meeting report. Lao PDR: Malaria Consortium, World Health Organization; 2011, 5-7 July.

[11] McCombie SC (1996). Treatment seeking for malaria: a review of recent research. Soc Sci Med.

[12] Williams HA, Jones CO (2004). A critical review of behavioral issues related to malaria control in sub-Saharan Africa: what contributions have social scientists made?. Soc Sci Med.

[13] ACT watch Group and PSI/Cambodia (2011). Household Survey Report (Endline).

[14] Aye SS, Nishino Y, Soe K, Zaw KK, Oo YTN, Han WW, et al. Role of private sector in Myanmar’s health care system: Implications for health sector reform. 2013. https://silo.tips/download/role-of-private-sector-in-myanmar-s-health-care-system-implications-for-health-s. Accessed 19 Dec 2021.

[15] The 2014 Myanmar Population and Housing Census. Department of Population MoL, Immigration and Population. Nay Pyi Taw 2014. p. 1-103

[16] Xu JW, Xu QZ, Liu H, Zeng YR (2012). Malaria treatment-seeking behaviour and related factors of Wa ethnic minority in Myanmar: a cross-sectional study. Malar J.

[17] Bennett A, Avancena ALV, Wegbreit J, Cotter C, Roberts K, Gosling R (2017). Engaging the private sector in malaria surveillance: a review of strategies and recommendations for elimination settings. Malar J.

[18] Hu J, Podhisita C (2008). Differential utilization of health care services among ethnic groups on the thailand-myanmar border: a case study of Kanchanaburi Province, Thailand. J Population Social Studies.

[19] Wang RB, Zhang J, Zhang QF (2014). Malaria baseline survey in four special regions of northern Myanmar near China: a cross-sectional study. Malar J.

[20] Township Profile _ Lashio Myanmar: General Administration Department, Ministry of Home Affairs.; 2017. http://themimu.info/township-profiles. Accessed 19 Dec 2021.

[21] Township Profile_ Kutkai Myanmar: General Administration Department, Ministry of Home Affairs.; 2017, http://themimu.info/township-profiles. Accessed 19 Dec 2021.

[22] Township Profile_ Kyaukme Myanmar: General Administration Department, Ministry of Home Affairs.; 2017. http://themimu.info/township-profiles. Accessed 19 Dec 2021.

[23] Township Profile_Hsipaw Myanmar: General Administration Department, Ministry of Home Affairs.; 2017. http://themimu.info/township-profiles. Accessed 19 Dec 2021.

[24] WHO. Regional Office for South-East Asia. Mobile and migrant populations and malaria information systems: World Health Organization; 2015. https://apps.who.int/iris/handle/10665/204343. Accessed 19 Dec 2021.

[25] StataCorp. Stata statistical software: release 14. College Station: StataCorp LP. 2015.

[26] ATLAS.ti 7 Windows: The computer-assisted qualitative data analysis software (CAQDAS). https://atlasti.com/. Accessed 19 Dec 2021.

[27] Linn SY, Maung TM, Tripathy JP, Shewade HD, Oo SM, Linn Z (2019). Barriers in distribution, ownership and utilization of insecticide-treated mosquito nets among migrant population in Myanmar, 2016: a mixed methods study. Malar J.

[28] Satitvipawee P, Wongkhang W, Pattanasin S, Hoithong P, Bhumiratana A (2012). Predictors of malaria-association with rubber plantations in Thailand. BMC Public Health.

[29] Humanitarian Needs Overview Myanmar: United Nations Office for the Coordination of Humanitarian Affairs; 2019. https://reliefweb.int/report/myanmar/myanmar-humanitarian-needs-overview-2020-december-2019. Accessed 19 Dec 2021.

[30] National strategic plan: Intensifying malaria control and accelerating progress towards malaria elimination (2016–2020). Myanmar: Department of Public Health, Ministry of Health and Sports, The Republic of the Union of Myanmar; 2016.

[31] Sonkong K, Chaiklieng S, Neave P, Suggaravetsiri P (2015). Factors affecting delay in seeking treatment among malaria patients along Thailand-Myanmar border in Tak Province. Thailand Malar J.

[32] Chaturvedi HK, Mahanta J, Pandey A (2009). Treatment-seeking for febrile illness in north-east India: an epidemiological study in the malaria endemic zone. Malar J.

[33] Nyamongo IK (2002). Health care switching behaviour of malaria patients in a Kenyan rural community. Soc Sci Med.

[34] Simsek Z, Kurcer MA (2005). Malaria: knowledge and behaviour in an endemic rural area of Turkey. Public Health.

[35] Community based health worker policy. Myanmar: Ministry of Health and Sports; 2020.

[36] Kheang ST, Lin MA, Lwin S, Naing YH, Yarzar P, Kak N (2018). Malaria case detection among mobile populations and migrant workers in Myanmar: comparison of 3 service delivery approaches. Glob Health Sci Pract.

[37] Verschuere J, Decroo T, Lim D, Kindermans JM, Nguon C, Huy R (2017). Local constraints to access appropriate malaria treatment in the context of parasite resistance in Cambodia: a qualitative study. Malar J.

